# Neonatal Microsurgical Repair of a Congenital Abdominal Aortic Aneurysm with a Cadaveric Graft

**DOI:** 10.1055/s-0041-1723019

**Published:** 2021-03-03

**Authors:** Annie Le-Nguyen, Shahrzad Joharifard, Geneviève Côté, Daniel Borsuk, Rafik Ghali, Michel Lallier

**Affiliations:** 1 Department of Surgery, Division of General Surgery, Saint Justine Hospital, Montreal, Quebec, Canada; 2 Department of Surgery, Division of Pediatric Surgery, Saint Justine Hospital, Montreal, Quebec, Canada; 3 Department of Anesthesiology, Saint Justine Hospital, Montreal, Quebec, Canada; 4 Department of Surgery, Division of Plastic Surgery, Saint Justine Hospital, Montreal, Quebec, Canada; 5 Department of Surgery, Division of Vascular Surgery, Hôpital Maisonneuve-Rosemont, Montreal, Quebec, Canada

**Keywords:** aortic aneurysm, abdominal, congenital, surgical repair, neonatal

## Abstract

Congenital abdominal aortic aneurysms (AAA) are an extremely rare entity. We present the case of a female fetus diagnosed with an AAA on routine prenatal ultrasound. A postnatal computed tomography angiogram revealed an infrarenal AAA with a narrow proximal neck. Surgery was performed on day of life 14 using a cadaveric femoral artery graft. The proximal anastomosis was performed under the microscope given the severity of the aortic stenosis and the proximity of the renal arteries. The patient's postoperative course was uneventful and she is developing normally 1 year after surgery. The graft remains permeable, albeit with evidence of proximal and distal stenosis and graft calcification on imaging.

## Introduction

Abdominal aortic aneurysms (AAA) rarely occur in infants and children. Acquired aneurysms are associated with connective tissue disorders, cardiac anomalies, autoimmune diseases, infection, trauma, or umbilical artery catheterization. 1 Congenital aneurysms, on the other hand, are idiopathic. 2 Since Howorth's original case reported in 1967, only 26 congenital AAAs have been documented, and among these, only seven were diagnosed antenatally. 3 4 5 Given the risk of spontaneous rupture, published case reports highlight the importance of prompt diagnosis and repair. To date, most surgeons have opted to use prosthetic grafts. 5 We report the case of an antenatally diagnosed congenital AAA extending from the renal arteries to the iliac bifurcation, which we repaired in the neonatal period using a cadaveric femoral artery graft and the aid of microsurgery.

## Case Report

The female fetus of a 29-year-old primigravida female was found to have a vascularized abdominal mass on prenatal ultrasound at 36 weeks' gestation. Following transfer to our quaternary center, a targeted fetal ultrasound confirmed an AAA measuring 3.1 × 2.3 cm AAA began at the level of the renal arteries and extended to the iliac bifurcation with a narrow proximal neck measuring just 1.7 mm. Fetal echocardiogram was normal and no signs of cardiac decompensation or fetal hydrops were noted. Parental questioning revealed no relevant family history. Prenatal consultations in neonatology, genetics, anesthesiology, pediatric surgery, and adult vascular surgery were obtained by the maternal–fetal medicine team. Following a multidisciplinary meeting, the decision was made to proceed with an induced vaginal delivery at 37 weeks.

**Fig. 1:**
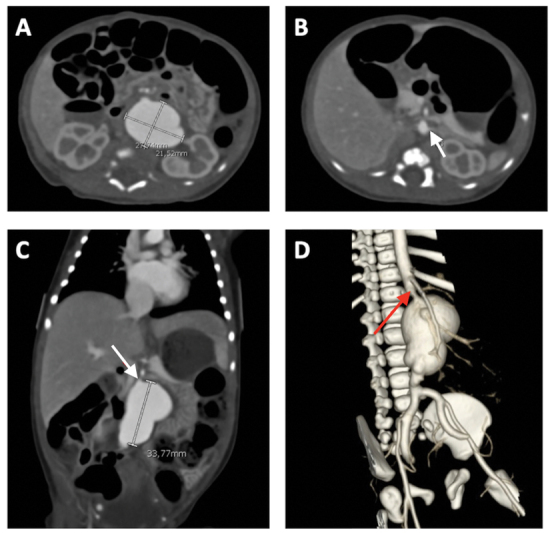
Initial computed tomography angiogram of chest, abdomen, and pelvis. Images ( **A–C** ) reveal a 2.8 × 3.3 × 2.2-cm congenital abdominal aortic aneurysm. Image ( **D** ) shows a three-dimensional reconstruction. Red arrows in images ( **C, D** ) denote the proximal 2-mm stenosis, while the white arrow in image ( **B** ) denotes the left renal artery.

The patient was born at 37 ^2/7^ weeks' gestation, weighing 2.78 kg. Fetal adaptation was uneventful. Abdominal exam was normal, without evidence of a pulsatile abdominal mass. Femoral pulses and inferior limb perfusion were normal. Umbilical artery catheterization was expressly avoided. Comparative genomic hybridization array and connective tissue gene tests did not identify a disease-causing mutation. Trans-fontanel cranial ultrasound and cardiac echocardiogram were normal. A computed tomography angiogram (CTA) of the chest, abdomen, and pelvis was obtained on day of life (DOL) 1, revealing an infrarenal, fusiform, and polylobate AAA measuring 2.8 × 3.3 × 2.2 cm ( Fig. 1 ). As would prove critical for operative planning, the CTA also revealed a 2-mm stenosis of the abdominal aorta at the level of the renal arteries, in addition to dilation of the common iliac artery and the internal iliac arteries.

The patient was kept in the neonatal intensive care unit and followed with serial Doppler ultrasounds, which on DOL 2 revealed a thrombus measuring 1.3 × 0.7 cm in the proximal portion of the aneurysm. Enoxaparin was initiated to prevent thrombus propagation and nifedipine was started to minimize risk of aneurysmal rupture. A subsequent ultrasound on DOL 4 revealed substantial thrombus growth to a size of 2.5 × 0.9 cm. Concern regarding further thrombus propagation or embolus, as well as near-certain mortality in the event of aneurysmal rupture, prompted an urgent multidisciplinary meeting to determine the optimal timing and approach for operative intervention.

Two major concerns were raised: (1) the high risk of anastomotic stenosis given the severe proximal aortic narrowing, and (2) the moderate risk of postoperative renal failure if insufficient space precluded infrarenal aortic clamping. To decrease the risk of proximal stenosis, we elected to use a 5-mm diameter cadaveric femoral artery graft obtained from a human tissue bank. In addition, building on our positive institutional experience with microsurgical anastomoses during pediatric liver transplantation, we decided to use microsurgery for the proximal anastomosis. Finally, we planned to place a hemodialysis catheter on the morning of surgery to allow for dialysis in the event of postoperative renal failure secondary to suprarenal clamping.

Surgery was performed on DOL 14. Noninvasive monitoring included a nasopharyngeal temperature probe, bladder catheter, and near-infrared spectroscopy (NIRS) neurologic monitoring (Somanetics Invos 5100C Cerebral/Somatic Oximeter for Regional Oxygen Saturation) using a cerebral probe and a renal probe. NIRS monitoring continually assessed the cerebral and infradiaphragmatic regional venous saturation. Invasive monitoring included two peripherally inserted central catheters, bilateral radial arterial lines, a right internal jugular hemodialysis catheter, and a transesophageal (TEE) probe (9T pediatric multiplane phase array probe, GE Healthcare). The core temperature was allowed to drift while the baby was installed and was subsequently maintained at 34°C during aortic clamping and as aortic perfusion was re-established. Rewarming was then initiated once adequate hemostasis was established.

**Fig. 2:**
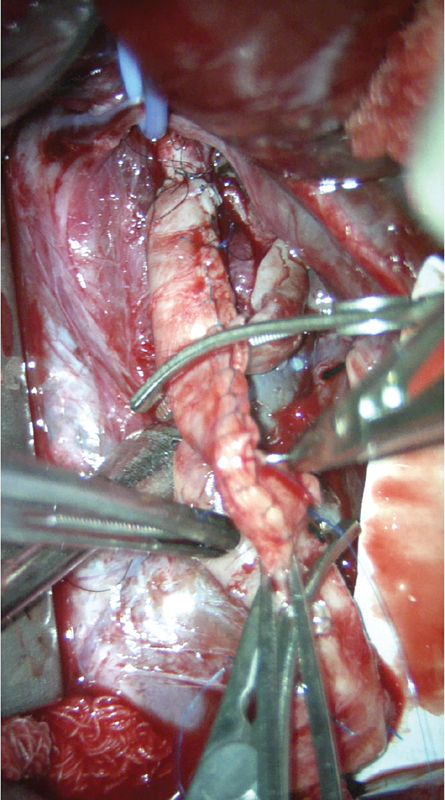
Intraoperative photograph taken using the microscope with the patient's head located toward the top of the image. The photograph shows the cadaveric graft, which was tapered ex vivo using a running suture of 7–0 polypropylene, as well as the proximal anastomosis, which was performed using interrupted 8–0 nylon to the stenotic native aorta. The blue vessel loop is around the inferior mesenteric artery.

The abdomen was explored via a midline incision from xiphoid to pubis. Despite preoperative concerns, there was adequate space to place an infrarenal aortic clamp. The aneurysmal husk was opened and the thrombus was evacuated. The graft was tapered using a running baseball stitch of 7–0 polypropylene (Prolene; Ethicon, United States). The proximal anastomosis was performed by a pediatric plastic surgeon (D.B.) using interrupted 8–0 nylon sutures under the microscope ( Fig. 2 ). Proximal clamp time was 100 minutes. The graft was then placed in a C-shape and anastomosed distally to the common iliac artery using a 7–0 polypropylene suture (Prolene; Ethicon, United States) with standard surgical loupes. The inferior mesenteric artery was ligated. Distal clamp time was 43 minutes. The aneurysmal husk was closed over the graft with a 5–0 polyglactin suture (Vicryl; Ethicon, United States) to decrease the risk of an aorto-duodenal fistula. The retroperitoneum was then closed with a 4–0 polyglactin suture (Vicryl; Ethicon, United States) followed by standard fascial and skin closure. Estimated blood loss was 206 mL, whereas her total blood volume was estimated at 216 mL.

The patient's postoperative course was largely unremarkable. Therapeutic heparin was initiated immediately, and aspirin was started on postoperative day (POD) 1. Following a transfusion of 20 cm ^3^ /kg of packed red blood cells for a drifting hemoglobin on POD 7, a contrast-enhanced CT was obtained, revealing a patent graft with expected proximal narrowing. Heparin was thus stopped and prophylactic enoxaparin reinitiated. The patient was discharged home on POD 26 with labetalol, pantoprazole, aspirin, and prophylactic enoxaparin.

The patient continues to do well 1 year after surgery with normal growth and palpable lower extremity pulses. Her most recent contrast-enhanced CT scan and Doppler ultrasound revealed a patent aortic graft with adequate flow; however, both studies showed proximal and distal stenosis, calcification within the graft, and development of vascular collateralization. The patient remains on low-molecular weight heparin and will continue to be followed with alternating CT and ultrasound imaging every 3 months.

## Discussion

Making the diagnosis of a congenital AAA first requires ruling out an acquired AAA. 6 Mycotic aneurysms are commonly caused by a traumatic umbilical artery catheterization or an infected or thrombosed umbilical arterial line. 7 Other causes of acquired AAA include vasculitis secondary to Kawasaki's syndrome, Takayasu's disease, polyarteritis nodosa, neurofibromatosis, or Bourneville's tuberous sclerosis. Aneurysms caused by connective tissue disorders such as Marfan or Ehlers–Danlos syndrome are caused by degeneration of the medial layer of the aorta but are more commonly discovered in the thoracic aorta and detected later in life between 4 and 15 years. 8 9 Finally, Loeys–Dietz syndrome, a connective tissue disorder related to mutations in the transforming growth factor β receptor is characterized by aggressive arterial aneurysms and aortic dissection in children and adolescents. 10

Congenital AAA are defined as localized truncular arterial defects, 11 but their etiology remains unclear. One relevant hypothesis is that congenital AAA result from a developmental defect during embryogenesis that creates a focal narrowing of the abdominal aorta, which leads to poststenotic turbulent blood flow and subsequent aneurysm formation. 2 Irrespective of etiology, congenital AAAs are rarely diagnosed on prenatal ultrasound. 12 13 14 They are more typically incidentally diagnosed after birth on ultrasound, CT, or MRI, 9 where they are occasionally misdiagnosed as abdominal cysts. 13 14 15 While there are no specific signs or symptoms, an AAA should be considered in a patient presenting with a pulsatile abdominal mass or abdominal murmur, especially if the patient is also hypertensive—a finding that can signal concomitant thrombosis of the renal artery. 2

The optimal management of congenital AAA remains undefined. Some authors have reported successful nonoperative management consisting of serial imaging, antiplatelet agents, and antihypertensive medications. Given that the risk of occlusion is high for grafts smaller than 6 mm, nonoperative management may be an option in stable patients to allow for the placement of a larger graft several months after birth. 16 A nonoperative approach can also be considered for patients in whom surgery conveys a high risk of mortality or significant complications, as is the case for aneurysms extending above the renal arteries or superior mesenteric artery take-off, aneurysms with severe proximal stenosis, or hemodynamic instability from concomitant medical problems. 17 If nonoperative management is pursued, patients must be followed with serial imaging to monitor for aneurysmal growth and the development of a mural thrombus, both of which predict impending rupture. 13 18 19 20 Indeed, Cribari et al found that 80% of patients who were treated nonoperatively died, and 42% of these deaths were caused by aneurysmal rupture. 21 Further, Mendeloff et al demonstrated that children undergoing surgical repair had a statistically significant higher probability of survival. 22

Given these statistics, the general consensus is that congenital AAAs should be surgically repaired, although several uncertainties remain. For one, the optimal timing of surgery is unclear. While operating early diminishes the risk of rupture, surgery in the neonatal period carries higher perioperative risk. In addition, the small diameter of a neonatal aorta poses a technical challenge in performing a nonstenotic vascular anastomosis. One solution is microsurgery, which has been shown to decrease rates of hepatic artery stenosis in the pediatric liver transplant population. 23 Moreover, if suprarenal clamping is required, there is significant risk for postoperative renal failure, and this can be difficult to manage since neonates do not tolerate hemodialysis well and peritoneal dialysis is not an immediate option following open abdominal surgery. Finally, optimal intraoperative anesthetic monitoring is uncertain, though our experience suggests that use of NIRS and TEE can assist in monitoring hemodynamic changes and optimizing responses to hemodynamic fluctuations that occur with clamping and unclamping.

As there are no published studies assessing graft patency, necessity for reoperation, or long-term survival, significant questions remain regarding optimal graft choice. Aneurysmorrhaphy, repair with native vessels, cryopreserved allografts, and various synthetic grafts have all been utilized to repair congenital AAAs. 24 Prosthetic grafts portend the risk of size mismatch between the graft and native vessel both at the time of surgery and as the child grows, in addition to long-term risks of infection and false aneurysm. 16 25 To decrease the need for reoperation, some have suggested the use of cryopreserved arterial grafts and C-shaped configuration of grafts. 26 We used a C-shaped cadaveric femoral artery graft to avoid the risk of infection and false aneurysm and to permit natural vessel growth. 14 Choosing a cadaveric graft will also allow for angiographic balloon dilation should the graft become stenotic or display insufficient growth as the child ages.

## Conclusion

Management of infants and children with congenital AAA requires a multidisciplinary approach. Thromboembolic events and rupture are potentially fatal outcomes of arterial aneurysms. Surgical repair is therefore recommended, but optimal timing and approach remain unclear given the lack of published long-term outcomes. There may be a theoretical advantage of using a C-shaped cadaveric artery graft as this choice will allow for growth and angiographic dilation over the patient's lifespan.

## Notes

New Insights and the Importance for the Pediatric Surgeon We report the first repair of a congenital abdominal aortic aneurysm (AAA) using a microsurgically anastomosed cadaveric femoral artery graft. While there is no standard approach to managing neonates with congenital AAAs, we conclude that a multidisciplinary strategy and careful perioperative planning are crucial in optimizing the treatment of these life-threatening vascular malformations.
