# Erratum to: Neonatal Microsurgical Repair of a Congenital Abdominal Aortic Aneurysm with a Cadaveric Graft

**DOI:** 10.1055/s-0041-1730902

**Published:** 2021-07-12

**Authors:** Annie Le-Nguyen, Shahrzad Joharifard, Geneviève Côté, Daniel Borsuk, Rafik Ghali, Michel Lallier

**Affiliations:** 1 Department of Surgery, Division of General Surgery, Saint Justine Hospital, Montreal, Quebec, Canada; 2 Department of Surgery, Division of Pediatric Surgery, Saint Justine Hospital, Montreal, Quebec, Canada; 3 Department of Anesthesiology, Saint Justine Hospital, Montreal, Quebec, Canada; 4 Department of Surgery, Division of Plastic Surgery, Saint Justine Hospital, Montreal, Quebec, Canada; 5 Department of Surgery, Division of Vascular Surgery, Hôpital Maisonneuve-Rosemont, Montreal, Quebec, Canada

Authors of the above-mentioned article DOI: 10.1055/s-0041-1723019 ; published online on March 3, 2021) have informed that the first two authors share equal authorship for the article. The information has been updated in the article.

